# The mutation rates of EGFR in non-small cell lung cancer and KRAS in colorectal cancer of Chinese patients as detected by pyrosequencing using a novel dispensation order

**DOI:** 10.1186/s13046-015-0179-9

**Published:** 2015-06-18

**Authors:** Guohua Xie, Fang Xie, Ping Wu, Xiangliang Yuan, Yanhui Ma, Yunchuan Xu, Li Li, Ling Xu, Ming Yang, Lisong Shen

**Affiliations:** Department of Clinical Laboratory, Xinhua Hospital, Shanghai Jiao Tong University School of Medicine, 1665 Kong Jiang Road, Shanghai, 200092 China; Department of General Surgery, Xinhua Hospital, Shanghai Jiao Tong University School of Medicine, Shanghai, 200092 China; Department of Anorectal Surgery and Colorectal Cancer Center, Xinhua Hospital, Shanghai Jiao Tong University School of Medicine, 1665 Kong Jiang Road, Shanghai, 200092 China

**Keywords:** EGFR, KRAS, NSCLC, CRC, FFPE sample, Pyrosequencing

## Abstract

**Background:**

The purpose of this study was to develop a cost-effective approach for the determination of EGFR and KRAS mutations in formalin-fixed paraffin-embedded (FFPE) non-small cell lung cancer (NSCLC) and colorectal cancer (CRC) samples from Chinese patients based on a sensitive pyrosequencing (PS) technique.

**Methods:**

The NSCLC and CRC cell lines were tested to determine the limitation of detection and reproducibility of the PS method. In addition, 494 NSCLC and 1099 CRC patient samples were assayed by PS to evaluate the EGFR or KRAS mutation patterns according to the clinicopathological features.

**Results:**

The PS assay was able to reproducibly detect as few as 2 % mutant alleles with excellent linearity. EGFR mutations were detected in 35.63 % of the NSCLC samples, and KRAS mutations were detected in 39.76 % of the CRC samples. EGFR mutations were more frequently observed to be significant by multivariate analysis in NSCLC patients who were 65 years old or younger (OR = 2.51), had a nonsmoking history (OR = 3.63), and adenocarcinoma (OR = 3.57), but not in females (OR = 0.64). KRAS mutations were more frequently detected in CRC patients who were female (OR = 1.64) and 50 years old or older (OR = 4.17), and had adenocarcinoma (OR = 2.41).

**Conclusions:**

This is the first extensive validation of PS on FFPE samples using the detection of EGFR exons 18–21 mutations and KRAS exon 2 mutations. Our results demonstrate the utility of PS analysis for the detection of somatic EGFR and KRAS mutations in clinical samples and provide important clinical and molecular characteristics of NSCLC and CRC from Chinese patients.

**Electronic supplementary material:**

The online version of this article (doi:10.1186/s13046-015-0179-9) contains supplementary material, which is available to authorized users.

## Introduction

Non-small cell lung cancer (NSCLC) and metastatic colorectal cancer (mCRC) are the most common cancers and the leading causes of cancer mortality [1]. Among the currently available therapeutic options, chemotherapy for NSCLC and mCRC has been only marginally effective. Therefore, the development of novel and effective therapy has been and continues to be imperative for public health. In the past decade, molecularly targeted therapeutics has been developed for the treatment of advanced NSCLC and mCRC. Some small molecule tyrosine kinase inhibitors (TKIs) and anti-epidermal growth factor receptor (EGFR) monoclonal antibodies (mAbs) have been shown to be effective both in preclinical and clinical trials. TKIs targeting a mutant EGFR and mAbs binding to the extracellular domain of EGFR have shown significant benefit in the clinic, but their efficacy depends on the mutations of EGFR in the tyrosine kinase region and wild-type V-Ki-ras2 Kirsten rat sarcoma viral oncogene homolog (KRAS) in the EGFR signaling pathway, respectively, which have become established predictive markers for the stratification of NSCLC and mCRC patients for targeted treatment [2, 3]. Accordingly, the US Federal Drug Administration has approved several TKIs and mAbs as anti-EGFR treatment for advanced NSCLC patients with the EGFR-activating mutation and for mCRC patients with wild-type KRAS, but not for NSCLC patients with wild-type EGFR or CRC patients with mutant KRAS [4, 5]. Thus, accurate incidence rates of EGFR and KRAS mutations are critical to reckon the effectiveness of molecular-targeted agents as personalized treatment for advanced NSCLC and mCRC patients in any given population.

During the past decade, the incidences of the EGFR mutation in NSCLC patients and the KRAS mutation in CRC patients have been shown to vary across different ethnicities. The mutation rate of EGFR in NSCLC patients is 16–18 % in North Americans and Europeans [6, 7], 19 % in African-Americans [8], 22 % in Indians [9], 29 % in Koreans [10], and 40 % in Japanese [11]; for the KRAS mutation in CRC patients, the rate is 37–54 % in North Americans and Europeans, 47 % in African-Americans [12], 24 % in Indians [13], 27 % in Koreans [12], and 38 % in Japanese [14], as summarized in Additional file 1: Table S1. However, data regarding the frequencies of EGFR and KRAS mutations in a Chinese population currently remain contradictory and confusing. The currently available data show that the frequency of NSCLC EGFR mutations in patients from mainland China varies from 19 to 56 % [9, 15–17], and the KRAS mutation rate in Chinese patients with CRC is 20–62 % [18–20]. Moreover, the incidence of EGFR and KRAS mutations might be underestimated or overestimated because of clinically selected cases and the small sample sizes in those studies. Thus, it is of high importance to accurately determine the mutation rates of EGFR in NSCLC and KRAS in CRC with a large cohort of patients.

In addition, with the advent of personalized medicine, there is an urgent need for routine methods for rapid and accurate detection of changes of nucleic acid in clinical specimens. A wide range of techniques exist for mutation detection, of which dideoxy sequencing has been the gold standard [21]. However, the limited sensitivity and long turnaround time of these available methods have prompted the development of alternative techniques for routine clinical testing that have greater diagnostic practicality [21, 22]. Pyrosequencing is one of the latest methods that uses luminometric instead of electrophoretic detection [23]. This technique enables characterization of mutations and quantification with high accuracy of mutated alleles in samples with a low tumor cell density. Pyrosequencing is particularly suitable for the targeted sequencing of short DNA fragments amplified from older and less optimal tissue samples [24]. Owing to its high sensitivity, pyrosequencing seems to present a more reliable approach, allowing rapid, accurate, and high-throughput detection of a minimal fraction of mutated cells in archived clinical tumor tissue [25].

In this study, we designed an accurate and reliable pyrosequencing assay to determine the EGFR mutation in 494 NSCLC patients and the KRAS mutation in 1099 CRC patients of Chinese ethnicity. We further retrospectively analyzed and correlated the EGFR and KRAS mutations across different variables including age, gender, smoking status, and histology groups.

## Materials and methods

### Cell lines and known mutated formalin-fixed paraffin-embedded (FFPE) tissues

Six human NSCLC and CRC cell lines (Additional file 1: Table S2) and four known EGFR and KRAS mutation-positive FFPE tumor tissues (Additional file 1: Table S3) were initially used to validate the accuracy of the established pyrosequencing method. All the cell lines were purchased from the Cell Bank of the Chinese Academy of Sciences (Shanghai, China). The four known mutation-positive FFPE tissues with an expected mutation profile based on previous Sanger sequencing analysis were histologically confirmed to be tumor cells according to the hematoxylin-eosin-stained slides.

### Collection of patient samples

NSCLC FFPE tissue specimens (*n* = 494) and CRC FFPE tissue specimens (*n* = 1099) were collected from the Department of Pathology of Xinhua Hospital affiliated to Shanghai Jiao Tong University School of Medicine (Shanghai, China) between January 2009 and December 2012. Written informed consent was obtained from all patients, and the study was approved by the Ethics Committee of Xinhua Hospital. Patient and tumor characteristics, such as age, gender, smoking status, histology and tumor sample type, are summarized in Table 1.

**Table 1:** Patient characteristics

Variable	Number (%)	Variable	Number (%)
NSCLC	494 (100)	CRC	1099 (100)
Age (years)		Age (years)	
Mean	64.70	Mean	60.95
Range	38–85	Range	36–82
Gender		Gender	
Male	334 (67.61)	Male	657 (59.78)
Female	160 (32.39)	Female	442 (40.22)
Smoking history		Sublocalization	
Ever smoker	348 (70.45)	Proximal colon	373 (33.94)
Never smoker	146 (29.55)	Distal colon	355 (32.30)
Pathological stage		Rectum	371 (33.76)
I	96 (19.43)	Dukes’ stage	
II	75 (15.18)	A	97 (8.83)
III	132 (26.72)	B	517 (47.04)
IV	185 (37.45)	C	343 (31.21)
Unknown	6 (1.21)	D	142 (12.28)
Pathology		Pathology	
ADC	310 (62.75)	ADC	1045 (95.09)
SCC	172 (34.82)	SCC	21 (1.91)
LCC	6 (1.21)	ASC	9 (0.82)
ASC	4 (0.81)	UDC	18 (1.64)
Others	2 (0.40)	Others	6 (0.55)
Differentiation		Differentiation	
Poor	97 (19.64)	Poor	256 (23.29)
Moderate	249 (50.40)	Moderate	752 (68.43)
Well	145 (29.35)	Well	78 (7.10)
Unknown	3 (0.61)	Unknown	13 (1.18)

*NSCLC* non-small cell lung cancer, *CRC* colorectal cancer, *ADC* adenocarcinoma, *SCC* squamous cell carcinoma, *LCC* large cell carcinoma, *ASC* adenosquamous carcinoma, *UDC* undifferentiated carcinoma

The material available for all tumors was tissue blocks. Before DNA extraction, representative sections were stained with haematoxylin and eosin (H&E) and tumors were reviewed by two pathologists (LS and MY) and histologically classified according to the 2004 WHO criteria. Moreover, the percentages of tumor cells and extracellular mucin, if there was a relevant amount (more than 50 % of the tumor), or lymphocyte inflammation (more than 10 % of lymphocytes at 20 × magnification) were assessed. Macrodissection was performed to guarantee at least 30 % tumor in all cases in which there was sufficient material available for analysis.

### DNA extraction

The genomic DNA of the human cell lines was extracted using the QIAamp DNA Mini Kit (Qiagen, Germany). The genomic DNA of the FFPE samples was extracted using the GTpure™ FFPE Tissue Kit (GeneTech, China). All of the extracted DNA samples were quantitated with NanoDrop spectrophotometer 2000c (Thermo Fisher, USA) and stored at −20 °C until use.

### Cell-mixing studies

To analyze the assay linearity and sensitivity, we performed reconstruction experiments with the NSCLC cell lines and CRC cell lines. The NSCLC or CRC cell lines harboring heterozygous or homozygous mutations were serially diluted with the corresponding wild-type cells. The proportions of the mutant cell lines were adjusted to 100 %, 50 %, 30 %, 20 %, 10 %, 5 %, 3 %, 2 %, and 0 % (mutant-type:wild-type), respectively. The genomic DNA extracted from each dilution was subjected to subsequent pyrosequencing and dideoxy sequencing on three separate, consecutive days. The actual percentage of the mutant allele was determined by pyrosequencing data from undiluted tumor cell DNA. For each tumor cell dilution, a theoretical percentage of the mutant allele was then calculated.

### Mutation analysis by pyrosequencing and direct dideoxy sequencing

The polymerase chain reaction (PCR) and pyrosequencing primers are listed in Additional file 1: Table S4. The PCR amplicons for pyrosequencing were bound to Streptavidin-Sepharose HP (GE Healthcare, USA), purified, washed, denatured with 0.2 M NaOH, and washed again. The pyrosequencing primer (0.3 μM) was annealed to the purified single-stranded PCR product, the pyrosequencing assay was performed on a PyroMark Q24 system (Qiagen, Germany), following the manufacturer’s instructions. The dispensation order for EGFR exons 18, 19, 20, and 21 was GCAAGTGCTGATGCTCGT, ACGAATACGACGACGCTAACAGTCTACGA, GACTAGCAGCTGCATGCT, and TGCGTGTCAACTACG, respectively. The dispensation order for the KRAS was TACGACTCAGATCGTAG. Our dispensation order was longer than that found in the commercial kit; additional bases were introduced in strategic positions, according to possible expected mutated sequences (Additional file 1: Figure S1). The amplicons for direct Sanger sequencing were analyzed and purified with the GTpure™ Gel/PCR Extraction Kit (GeneTech), and sequenced on an ABI 310 genetic analyzer (Applied Biosystems).

### Statistical analysis

All statistical analyses were performed using SAS ver. 9.2 (SAS Institute, Cary, NC). For continuous variables, the median and range were calculated. A *χ*^2^ test was performed in a univariate analysis in order to investigate the relationship between the EGFR or KRAS gene mutation rate and patient background factors. Based on the results, multivariate logistic regression was performed, and the odds ratio (OR) and 95 % confidence interval (95 % CI) were calculated. Statistical significance was set at *p* < 0.05.

## Results

### Pyrosequencing with novel nucleotide dispensation order accurately detects EGFR or KRAS mutations both in cultured cells and FFPE tissues

**Fig. 1:**
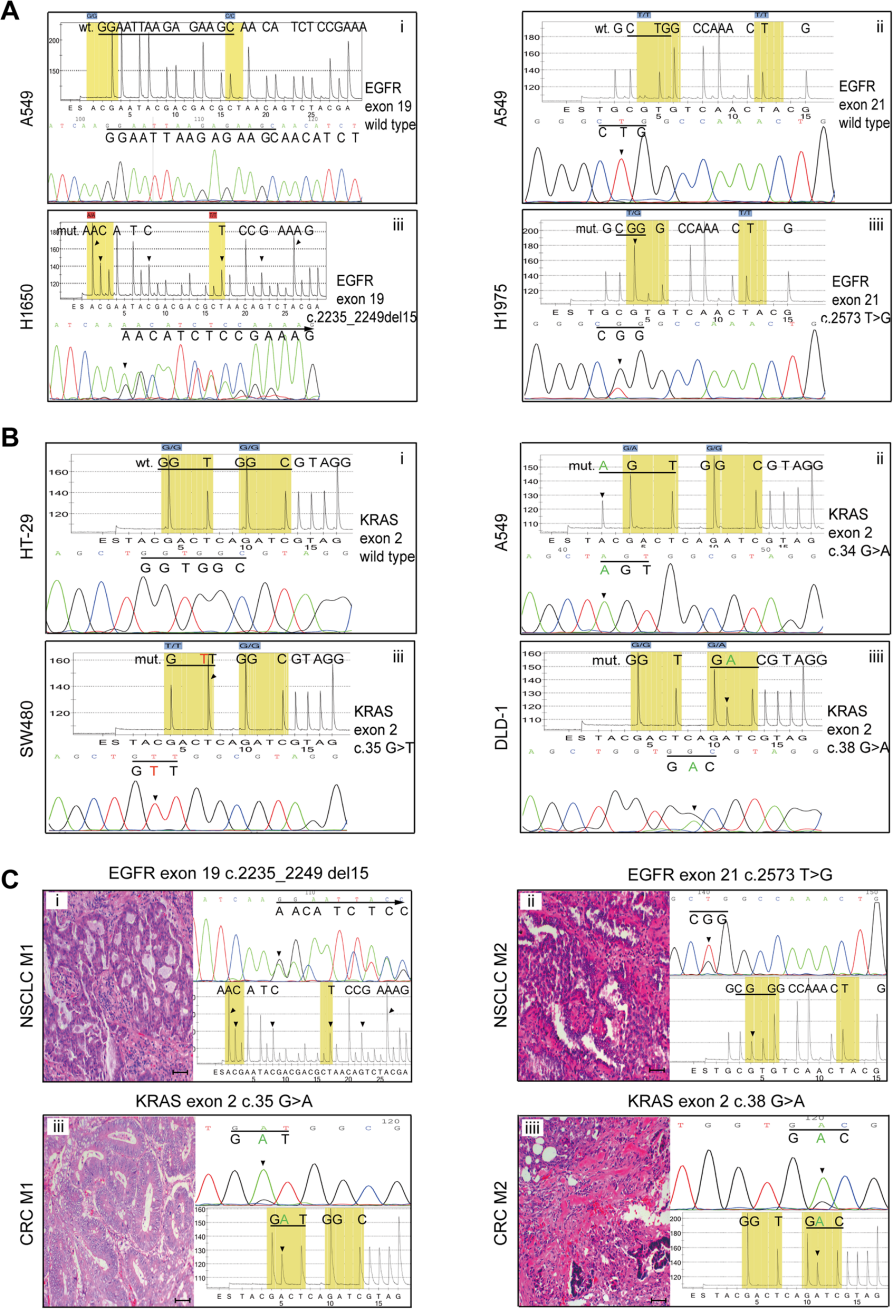
Validation of the established pyrosequencing analysis method using a novel dispensation order on cell lines and FFPE tissues. DNA from six tumor cell lines and four FFPE tissues was analyzed in parallel by pyrosequencing and dideoxy sequencing. **a** Examples of pyrograms obtained on the NSCLC cell lines A549 (*i* and *ii*), H1650 (*iii*), and H1975 (*iiii*) for mutational analysis of EGFR exon 19 or exon 21. **b** Examples of pyrograms obtained on the CRC cell lines HT-29 (*i*), SW480 (*iii*), and DLD-1 (*iiii*) as well as the NSCLC cell line A549 (*ii*) for mutational analysis of KRAS exon 2, codons 12 and 13. **c** To test the suitability of pyrosequencing for clinical samples, the assay was used to analyze EGFR or KRAS mutations in the following known mutated FFPE tissues: NSCLC M1 (*i*), NSCLC M2 (*ii*), CRC M1 (*iii*), and CRC M2 (*iiii*). All of the targeted mutations occurring in the cell lines and FFPE tumor tissues could easily be distinguished by pyrosequencing. The results for the six cell lines and four FFPE tissues were 100 % concordant between the established pyrosequencing and dideoxy sequencing methods. The target nucleotide sequences are labeled and underlined. The horizontal axis of each pyrogram, from left to right, indicates the order of reagent addition. E represents enzyme. S represents substrate. Mutation points are indicated by light shading. The vertical axis represents the luminescence intensity, of which the peak heights are proportional to the number of each nucleotide incorporated at one time. Sites of variation are indicated by arrows. Data are representative of five independent analyses of the same sample

The established pyrosequencing method was able to discriminate the wild-type from the different mutations presented in the mutant cell lines. As seen in the pyrosequencing traces of the targeted sequence of the six cell lines, the NSCLC cell lines H1650 (c.2235_2249del15, Fig. 1a*iii*) and H1975 (c.2573T>G, Fig. 1a*iiii*) showed specific peak patterns, which were readily distinguishable from that of the EGFR exons 19 and 21 wild-type NSCLC cell line A549 (Fig. 1a*i* and *ii*). Similarly, the A549 (KRAS exon 2 c.34G>A, Fig. 1b*ii*) and the CRC cell lines SW480 (KRAS exon 2 c.35G>T, Fig. 1b*iii*) and DLD-1 (KRAS exon 2 c.38G>A, Fig. 1b*iiii*) showed specific pyrogram traces, which were readily distinguishable from that of the KRAS exon 2 wild-type CRC cell line HT-29 (Fig. 1b*i*). The designed pyrosequencing assay was further tested with the known mutant NSCLC- and CRC-FFPE tissues (Additional file 1: Table S3). The two types of NSCLC-FFPE tissues harboring different EGFR TK domain mutations (Fig. 1c*i* and *ii*) and the two types of CRC-FFPE tissues with known KRAS exon 2 mutations (Fig. 1c*iii* and iiii) were detected by the established pyrosequencing analysis, respectively. Dideoxy sequencing confirmed the results of the pyrosequencing traces.

### Pyrosequencing with novel nucleotide dispensation order demonstrates high sensitivity and excellent linearity and reproducibility in detecting EGFR and KRAS mutations

**Fig. 2:**
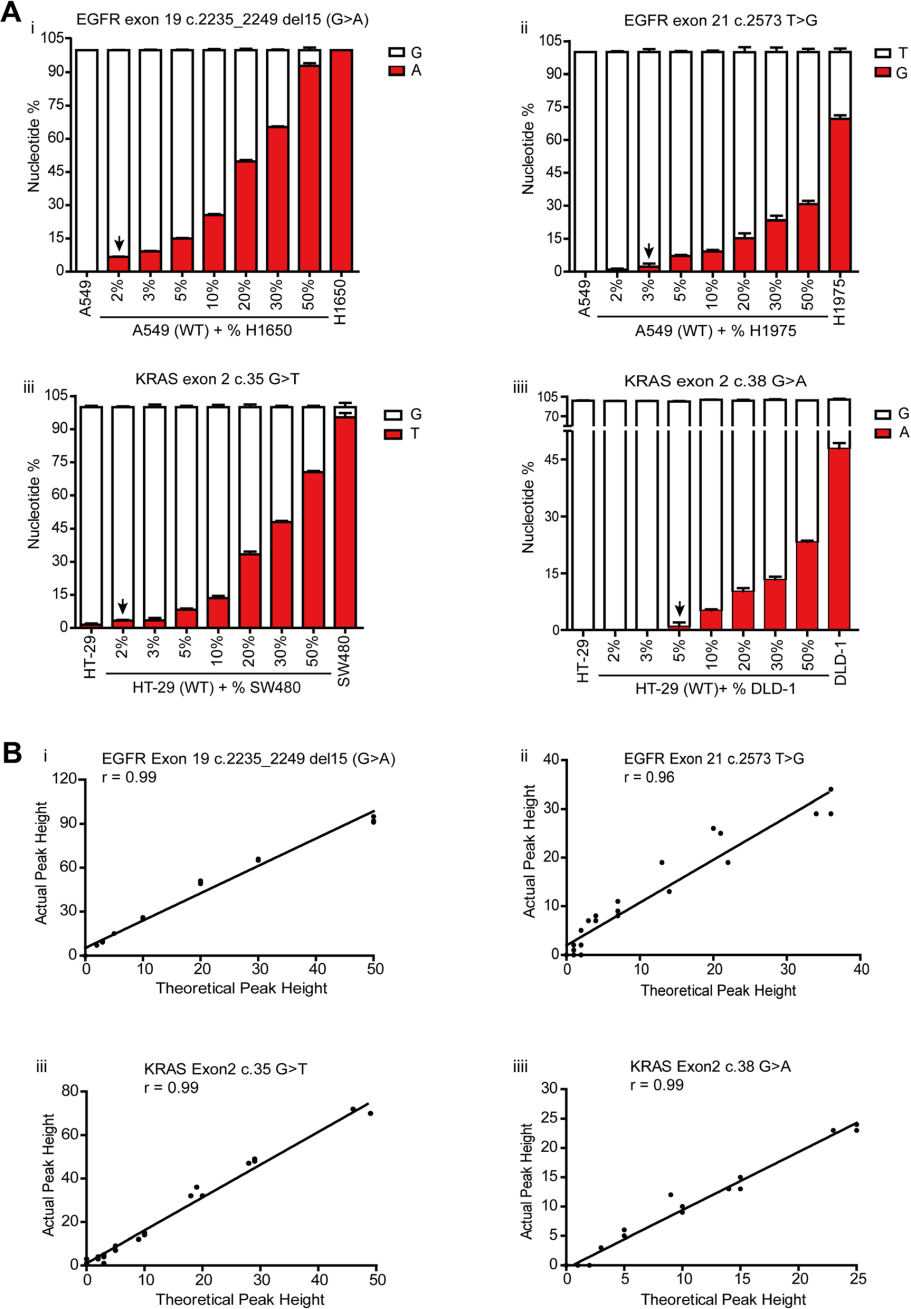
Limit of detection and assay linearity. Mixtures of DNA from EGFR or KRAS cell lines were analyzed in triplicate and in parallel by pyrosequencing and dideoxy sequencing methods to analyze the limit of detection and assay linearity (Additional file 1: Figures S2–S5, Tables S5–S6). **a** To assess the limit of detection, cell line DNA was mixed to produce samples containing differing proportions of mutant alleles. The established pyrosequencing method allows for 2 % of EGFR exon 19 c.2235_2249del15 mutant alleles (*i*), 3 % of EGFR exon21 c2573 T>G mutant alleles (*ii*), 2 % of KRAS exon2 c.35G>T mutant alleles (*iii*), and 5 % of KRAS exon 2 c.38G>A mutant alleles (*iiii*) to be detected with confidence. The percentages on the horizontal axis indicate the calculated percentages of mutant alleles present, while the vertical axis indicates the percentage of nucleotide. The white column represents the percentage of detected wild-type nucleotides; the red column within the white column represents the percentage composition of the detected mutant nucleotides. Arrows indicate the DNA dilutions in which mutations could be reliably detected above background noise. WT means wild-type. **b** To assess the assay linearity of pyrosequencing, theoretical peak heights, calculated from the initial mutant allele peak percentage in undiluted mutant cells, were correlated with actual peak heights generated for each dilution. For mutational analysis of EGFR exon 19 c.2235_2249del15 (*i*), KRAS exon 2 c35 G>T (*iii*), and c.38 G>A (*iiii*), Pearson’s correlation (r) = 0.99; for mutational analysis of EGFR exon 21 c.2573 T>G (*ii*), r = 0.96, indicating a linear relationship. A, adenine; G, guanine; T, thymine; C, cytosine. Data are from three independent experiments

Assay sensitivity of the pyrogram profile in discriminating different percentages of mutated alleles was initially evaluated by using serial dilutions of mutated DNA, derived from a cultured cell line with a known EGFR or KRAS mutation, and variably mixed with wild-type DNA obtained from a wild-type cell line. We tested 100 %, 50 %, 30 %, 20 %, 10 %, 5 %, 3 %, 2 %, and 0 % (mutant-type/wild-type) dilutions of H1650, H1975, SW480, and DLD-1cell lines (Fig. 2a). Our results demonstrated that this method could detect at least 2 % of the mutated alleles in the heterozygous mutant EGFR c.2235_2249del15 (Fig. 2a*i*) and homozygous mutant KRAS c.35G>T (Fig. 2a*iii*), 3 % of the mutated alleles in the heterozygous mutant EGFR c.2573T>G dilution (Fig. 2a*ii*), and 5 % of the mutated alleles in the heterozygous mutant KRAS c.38G>A dilution (Fig. 2a*iiii*). Compared with the dideoxy sequencing tracings from the amplifications on day 1, a small mutant peak was visible in the dideoxy sequencing tracings of the 15–20 % homozygous to 20–30 % heterozygous mutant dilutions (Additional file 1: Figures S2–S5).

For linearity testing, based on the data generated from the separate NSCLC or CRC cell mixing analyses, a linear relationship between the actual and theoretical percentages of the mutant allele was identified (Fig. 2b, Additional file 1: Tables S5-S6). The coefficient of correlation (r) was 0.99 for EGFR exon 19 c.2235_2249del15 (G>A) (Fig. 2b*i*), KRAS exon 2 c.35G>T (Fig. 2b*iii*), and KRAS exon 2 c.38G>A (Fig. 2b*iiii*); the r value was 0.96 for EGFR exon 21 c.2573 T>G (Fig. 2b*ii*), indicating an excellent linearity and reproducibility.

### Pyrosequencing analysis results of EGFR mutations in exons 18–21 in 494 NSCLC clinical samples

After standardization and validation by direct DNA sequencing for the robustness, linearity, and sensitivity of our designed pyrosequencing method, we applied it as a routine clinical test for common EGFR mutation assay in 494 patients diagnosed with NSCLC (Table 1). Overall, 35.63 % (176/494) of the NSCLC samples were identified to have EGFR TK domain mutations, representing 16 different nucleotide mutation types (Additional file 1: Figure S6). A total of 18.83 % (93/494) of the mutations were located in exon 19, and 16.80 % (83/494) were located in exon 21. A total of 52.27 % (92/176) were small in-frame deletions in exon 19, while 47.16 % (83/176) were missense mutations in exon 21. The most common mutation in exon 19 was delE746-A750 (56.99 %, 53/93). In exon 21, the most common mutation was located on L848R (97.59 %, 81/83). One rare exon 19 missense mutation (c.2239 TT>CC, p.L747P) was detected. EGFR exon 18 and exon 20 mutations were not detected in this cohort of NSCLC samples (Table 2).

**Table 2:** Summary of EGFR exons 18–21 mutations in 494 NSCLC-FFPE tissues and KRAS exon 2 mutations in 1099 CRC-FFPE tissues as detected by our designed pyrosequencing method

Gene	Exon	Type of mutation	Nucleotide change	Amino acid change	No. with mutation (%)
EGFR	19	Missense	c.2239TT>CC	p.L747P	1 (0.20)
	19	Deletion	c.2235_2249del15	p.E746_A750	53 (10.73)
			c.2236_2250del15	p.E746_A750	18 (3.64)
			c.2237_2251del15	p.E746_T751	2 (0.40)
			c.2237_2253del17	p.E746_T751	1 (0.20)
			c.2237_2255del19	p.E746_S752	1 (0.20)
			c.2237_2257del21	p.E746_P753	1 (0.20)
			c.2239_2248del10	p.L747_E749	2 (0.40)
			c.2239_2253del15	p.L747_T751	1 (0.20)
			c.2239_2256del18	p.L747_S752	2 (0.40)
			c.2240_2254del15	p.L747_T751	2 (0.40)
			c.2240_2257del18	p.L747_P753	5 (1.01)
			c.2253_2276del24	p.S752_I759	3 (0.61)
			c.2254_2277del24	p.S752_I759	1 (0.20)
	21	Missense	c.2573T>G	p.L858R	81 (16.40)
			c.2582T>A	p.L861Q	2 (0.40)
Total					176 (35.63)
KRAS	2	Missense	c.34G>T	p.G12C	24 (2.18)
			c.34G>A	p.G12S	26 (2.37)
			c.34G>C	p.G12R	1 (0.09)
			c.35G>A	p.G12D	168 (15.29)
			c.35G>C	p.G12A	23 (2.09)
			c.35G>T	p.G12V	85 (7.73)
			c.34G>A + c.35G>A	p.G12S + p.G12D	6 (0.55)
			c.37G>T	p.G13C	4 (0.36)
			c.38G>A	p.G13D	100 (9.10)
Total					437 (39.76)

*EGFR* epidermal growth factor receptor, *KRAS* V-Ki-ras2 Kirsten rat sarcoma viral oncogene homolog, *NSCLC* non-small cell lung cancer, *CRC* colorectal cancer, *FFPE* formalin-fixed paraffin-embedded

### EGFR mutations and clinicopathological features of NSCLS patients

Using univariate analysis, when all histologies were analyzed together, females showed a significantly higher frequency of the EGFR mutation compared with males (42.50 % vs. 32.34 %, *p* = 0.028). Within the different age groups, the mutation rate was significantly higher in those 65 years old or younger than in those older than 65 years old (45.02 % vs. 24.22 %, *p* < 0.001). Those with ADC showed higher mutations than any other histology (45.48 % vs. 19.02 %, *p* < 0.001). When the ADC subgroup was analyzed by gender, the mutation rate was predominantly higher among females compared to males (56.12 % vs. 40.57 %, *p* = 0.011). In addition, the smoking status was closely associated with the EGFR mutation rate, and the mutation rate was higher in the never-smoker group than in the smoker group (50.68 % vs. 29.31 %, *p* < 0.001 (Table 3). However, there was no difference in the EGFR mutation rate between males and females in the never-smoker group (52.38 % vs. 50.40 %, *p* = 0.867). Similarly, no significant correlation of EGFR mutations between the never-smoker male or female ADC patients (60.00 % vs. 65.38 %, *p* = 0.69) was observed. When sex, age, histotype, and smoking history were tested by multivariate analysis against the presence of EGFR mutations as a dependent variable, only the female data did not remain significant. No significant associations between EGFR mutations and differentiation status or tumor stage were observed (Table 3).

**Table 3:** Correlation of EGFR mutations with clinicopathological features of NSCLC patients

Variable	Number (%)	EGFR		Univariate		Multivariate	
		Mutation (%)	Wild type (%)	Odds ratio (95 % CI)	*p* value	Odds ratio (95 % CI)	*p* value
Gender							
Male	334 (67.61)	108 (32.34)	226 (67.66)	Ref.		Ref.	
Female	160 (32.39)	68 (42.50)	92 (57.50)	1.55 (1.05–2.28)	0.028	0.64 (0.32–1.28)	0.209
Age							
≤65	271 (54.86)	122 (45.02)	149 (54.98)	2.56 (1.73–3.78)	<0.001	2.51 (1.65–3.81)	<0.001
>65	223 (45.14)	54 (24.22)	169 (75.78)	Ref.		Ref.	
Smoking history							
Ever smoker	348 (70.45)	102 (29.31)	246 (70.69)	Ref.		Ref.	
Never smoker	146 (29.55)	74 (50.68)	72 (49.32)	2.48 (1.66–3.69)	<0.001	3.63 (1.83–7.19)	<0.001
Never smoker							
Male	21 (14.38)	11 (52.38)	10 (47.62)	Ref.			
Female	125 (85.62)	63 (50.40)	62 (49.60)	0.92 (0.37–2.33)	0.867		
Pathologic stage							
I + II	171 (34.62)	55 (32.16)	116 (67.84)	Ref.		Ref.	
III + IV	323 (65.38)	121 (37.46)	202 (62.54)	1.26 (0.85–1.87)	0.243	1.45 (0.94– 2.24)	0.089
Pathology							
Non-ADC	184 (32.75)	35 (19.02)	149 (80.98)	Ref.		Ref.	
ADC	310 (62.75)	141 (45.48)	169 (54.52)	3.55 (2.31–5.46)	<0.001	3.57 (2.28–5.61)	<0.001
Male	212 (68.39)	86 (40.57)	126 (59.43)	Ref.			
Female	98 (31.61)	55 (56.12)	43 (43.88)	1.87 (1.15–3.04)	0.011		
Never smoker ADC							
Male	15 (16.13)	9 (60.00)	6 (40.00)	Ref.			
Female	78 (83.87)	51(65.38)	27(34.62)	1.26 (0.41–3.91)	0.69		
Differentiation							
Well	145 (29.35)	50 (34.48)	95 (65.52)	Ref.		Ref.	
Moderate	249 (50.40)	92 (36.95)	157 (63.05)	1.11 (0.73–1.71)	0.623	1.14 (0.72–1.82)	0.573
Poor	97 (19.64)	34 (35.05)	63 (64.95)	1.03 (0.60–1.76)	0.927	1.10 (0.60–2.00)	0.759

*EGFR* epidermal growth factor receptor, *NSCLC* non-small cell lung cancer, *ADC* adenocarcinoma, *CI* confidence interval, *Ref.* reference group

### Pyrosequencing analysis results of KRAS mutations in exon 2 in 1099 CRC clinical samples

In 1099 Chinese CRC-FFPE samples (Table 1), a total of 437 missense mutations were detected at KRAS codons 12 and 13 (39.76 %, 437/1099). Nine types of KRAS exon 2 hotspot mutations were observed (Additional file 1: Figure S7). The mutation frequencies of exon 2, codon 12 and codon 13 were 30.30 % (333/1099) and 9.46 % (104/1099), respectively. The frequently observed mutations were G>A transition (300/437, 67.65 %), followed by G>T transversion (113/437, 25.86 %), and G>C transversion (24/437, 5.49 %). Furthermore, 76.20 % (333/437) of the mutations were located in codon 12, in which the base substitutions were mainly located at the first and second nucleotides. The screening results also showed that six CRC patients harbored codon 12 double G>A transitions (c.34G>A and c.35G>A, 1.37 %, 6/437). Meanwhile, 104 patients had detectable mutations at codon 13 (23.80 %, 104/437), and the G>A transition at the second nucleotide was the most frequent mutant type (c.38G>A, 96.15 %, 100/104) (Table 2).

### KRAS mutations and clinicopathological features of CRC patients

By univariate analysis, correlation of the KRAS mutation with clinicopathological data indicated that KRAS mutations were higher in females than in males (46.15 % vs. 35.46 %, *p* < 0.01) (Table 4). Interestingly, KRAS mutations were significantly higher in patients who were older than 50 years old in comparison with patients younger than 50 years old (45.67 % vs. 17.67 %, *p* < 0.001), indicating that KRAS mutations are uncommon in younger CRC patients. Moreover, KRAS mutations were more prevalent in ADC patients when compared to non-ADC patients such as those with SCC, ASC, and UDC (40.86 % vs. 18.52 %, *p* = 0.002). However, KRAS mutations were not found to be significantly associated with moderately/poorly differentiated tumors in comparison with well-differentiated tumors. The logistic multivariate analysis results were in line with the univariate analysis results. No significant association between KRAS mutations and tumor sublocalization or the Dukes’ stage was observed in these CRC patients (Table 4).

**Table 4:** Correlation of KRAS mutations with clinicopathological features of CRC patients

Variables	Number (%)	KRAS		Univariate		Multivariate	
		Mutant (%)	Wild-type (%)	Odds ratio (95 % CI)	*p* value	Odds ratio (95 % CI)	*p* value
Gender							
Male	657 (59.78)	233 (35.46)	424 (64.54)	Ref.		Ref.	
Female	442 (40.22)	204 (46.15)	238 (53.85)	1.56 (1.22–1.99)	<0.001	1.64 (1.26–2.12)	<0.001
Age (years)							
≤50	232 (21.11)	41 (17.67)	191 (82.33)	Ref.		Ref.	
>50	867 (78.89)	396 (45.67)	471 (54.33)	3.92 (2.72–5.63)	<0.001	4.17 (2.85–6.10)	<0.001
Sub-localization							
Rectum	371 (33.76)	139 (37.47)	232 (62.53)	Ref.		Ref.	
Prioximal colon	373 (33.94)	155 (41.55)	218 (58.45)	1.19 (0.88–1.59)	0.254	0.85 (0.62–1.17)	0.333
Distal colon	355 (32.30)	143 (40.28)	212 (59.72)	1.13 (0.84–1.52)	0.437	0.95 (0.69–1.31)	0.754
Dukes’ stage							
A + B	614 (56.23)	244 (39.74)	370 (60.26)	Ref.		Ref.	
C + D	478 (43.77)	192 (40.17)	286 (59.83)	1.02 (0.80–1.30)	0.886	0.99 (0.77–1.29)	0.963
Pathology							
Non-ADC	54 (4.91)	10 (18.52)	44 (81.48)	Ref.		Ref.	
ADC	1045 (95.09)	427 (40.86)	618 (59.14)	3.04 (1.51–6.11)	0.002	2.41 (1.12–5.17)	0.024
Differentiation							
Well	78 (7.10)	23 (29.49)	55 (70.51)	Ref.		Ref.	
Moderate	752 (68.43)	310 (41.22)	442 (58.78)	1.68 (1.01–2.79)	0.046	1.10 (0.62–1.96)	0.742
Poor	256 (23.29)	101 (39.45)	155 (60.55)	1.56 (0.90–2.69)	0.112	1.24 (0.66–2.30)	0.503
Unknown	13 (1.18)	3 (23.08)	10 (76.92)	0.72 (0.18–2.85)	0.637	0.53 (0.13–2.24)	0.388

*KRAS* V-Ki-ras2 Kirsten rat sarcoma viral oncogene homolog, *CRC* colorectal cancer, *CI* confidence interval, *ADC* adenocarcinoma, *Ref.* reference group

## Discussion

In the present study, we established a practical and reliable pyrosequencing assay using novel nucleotide dispensation order for the detection of EGFR and KRAS mutations in a large cohort of Chinese NSCLC and CRC patients. We further retrospectively analyzed the actual incidence of genetic abnormalities of EGFR and KRAS and their distribution according to clinicopathological features in Chinese patients.

In our analysis of 494 NSCLC cases, the EGFR mutation rate was 35.63 % in the Chinese population, which was significantly higher than the EGFR mutation rates reported in western countries, but was similar to that of other Eastern populations (Additional file 1: Table S1). We found a higher rate of the EGFR mutation in ADC patients than in all patients (45.48 % vs. 35.63 %) and among females compared to males (42.50 % vs. 32.34 %), similar to rates in Indian, Korean, and Japanese patients [10, 11, 26]. Although the mutation rate varies significantly between ever smokers and never smokers across different ethnicities, EGFR mutations have been consistently reported to be more common in never smokers as compared to ever smokers [9–11, 16, 27]. Consistently, we found that the mutation rates of EGFR for ever smokers and never smokers were 50.68–29.31 %, respectively. Histopathologically, the mutation rates among females with ADC were predominantly higher than in males with ADC (56.12 % vs. 40.57 %), consistent with previous studies [28, 29]. However, no significant differences were observed between never-smoker ADC males and females, indicating that the lack of a gender bias among never smokers was possibly due to the fact that there was a lower proportion of nonsmoking males (21 nonsmokers out of 334 males) than nonsmoking females (125 nonsmokers out of 160 females) in this study. These findings are also consistent with similar studies on females of other Asian ethnicities who never smoked, wherein the EGFR mutation rate varies with the clinical stage of the female never-smoker patients [30–32]. For lung SCC, there have been few studies focusing on Asians, with conflicting results. Previous studies have shown that the EGFR mutation rates of South Korean, Indian, and Japanese patients were 0.96 % (1/104), 3.88 % (4/103), and 6.50 % (8/123) [33–35], respectively. Two Chinese-based studies reported EGFR mutation rates of 14.54 % (41/282) and 29.73 % (11/37) [16, 17]. In the SCC cohort of this study (*n* = 172), we found that the EGFR mutation rate was 19.19 % (33/172), similar to the previously reported Chinese-based data, implying that the EGFR mutation test for SCC needs to be considered as a routine practice.

Our retrospective analysis of 1099 samples from Chinese CRC patients showed that the frequency of a KRAS mutation was 39.76 %, which is similar to the previously published data from Japan, the Netherlands, Germany, and the United States (37–43 %), but higher than those from Thailand, India, South Korea, Oman, and Australia (23–31 %) (Additional file 1: Table S1). Except for the consideration of the sensitivity of the techniques used in some studies, the variations in the frequency of the KRAS mutation worldwide may be explained by the patient ethnicity and geographical distribution. Studies from western nations have reported KRAS G12D as the most recurrent transition, followed by G12V, G12C, G12S, and G12A [36, 37]. In our study, the corresponding order was G12D, G12V, G12S, G12C, and G12A. Among KRAS codon 13 mutations, G13D was the major mutation, followed by G13C and G13R or G13S in western populations [36, 37]. However, in the current study, predominantly only G13D and rarely G13C mutations were observed, while none of the cases showed G13R. These data suggest that there may be some racial differences in the patterns of KRAS mutations.

In agreement with previous studies [14, 19, 38], our results showed that KRAS mutations occurred more frequently in females than in males. Moreover, consistent with studies of India and Japan [13, 14], CRC patients older than 50 years old demonstrated significantly higher KRAS mutation rates than patients younger than 50 years old (396/867), indicating that the older the patient, the higher the KRAS mutation rate in CRC patients. Interestingly, our findings demonstrate that tumors with KRAS mutation tend to occur more frequently in ADC as compared to the non-ADC subtypes, which further supports the previous observation [13]. Other clinicopathological features including tumor differentiation, location, and staging did not show any association with KRAS mutation in our retrospective analysis.

Regarding the importance of EGFR and KRAS mutation detection for the prediction and prognosis of NSCLC and CRC, a reliable diagnostic test may affect future therapeutic decision-making. In this study, we demonstrated that our designed pyrosequencing assay using a novel nucleotide dispensation order is a reliable and accurate technique for the detection of EGFR and KRAS hotspot mutations from FFPE tissues. First, pyrosequencing was established and validated in a set of DNA samples obtained from several cell lines and mutation-positive FFPE tumor tissues. The homozygous or heterozygous mutations in the NSCLC (Fig. 1a) and CRC cell lines (Fig. 1b) and mutant FFPE tissues (Fig. 1c) were accurately detected with this pyrosequencing method. Next, the sensitivity for detection of mutations by our designed pyrosequencing method was sufficiently high as it was able to detect mutations in samples containing as few as 2 % homozygous to 5 % heterozygous mutated alleles in a background of wild-type DNA (Fig. 2a). Pearson’s (r) value for the four detected mutation types statistically validated the excellent assay linearity and reproducibility (Fig. 2b). In addition, this method was applied to detect mutations in a large series of FFPE tissues and compared with dideoxy sequencing; the results showed a high overall agreement with genotyping, with more mutations detected by the pyrosequencing assay (data not shown).

Dideoxy sequencing and real-time PCR-based techniques are currently the most commonly performed methods for the detection of EGFR and KRAS mutations in the clinic. Direct DNA sequencing has several disadvantages with regard to clinical application [20–22]. The most notable one is its requirement for a DNA template with a relatively high quality. Moreover, it has a relatively low sensitivity, as also revealed by this study (Additional file 1: Figures S2–S5). The real-time PCR-based approaches, such as the amplification refractory mutation system, peptide nucleic acid/locked nucleic acid clamp PCR, and coamplification at lower denaturation temperature PCR have been reported to be more sensitive and detect as low as 1 % mutated alleles in samples containing a mixture of tumor and normal cells [16, 22]; however, the results obtained are restricted to the screening of mutant versus wild-type tumors and lack any further characterization. In contrast, pyrosequencing could detect all of the mutations within the amplified region, show DNA sequences around targeted nucleotide(s), and provide the quality assurance measurement that is especially important in clinical settings [21, 39]. In this study, our designed pyrosequencing assay using a novel dispensation order could detect and characterize both classical and uncommon EGFR and KRAS mutations (Additional file 1: Figures S1, S6, and S7). In addition, most of the types of degraded DNA extracted from NSCLC- or CRC-FFPE tissues could be used as the pyrosequencing template directly, without requiring further dilution or normalization. Thus, compared with other techniques, the pyrosequencing assay described herein is relatively simple, reliable, and fast. Although real-time PCR-based approaches seem to be more sensitive than our modified pyrosequencing assay, the sensitivity for the detection of mutations by this method is sufficient as it could detect mutations in samples containing as few as 2 % mutated cancer cells (Fig. 2a).

There were some limitations in this retrospective study. First, we had no data on survival analysis. Second, we were unable to demonstrate the intra-patient variability of the EGFR or KRAS mutations between primary lesions and metastatic lesions, because the tests for EGFR and KRAS mutations were performed in either the primary tumor or metastatic tumor sample from each patient. Despite these limitations, using this novel pyrosequencing method in routine clinical practice provided a large-scale screening that included unselected Asian NSCLC and CRC patients. Furthermore, it demonstrated the prevalence of EGFR and KRAS mutations and mutation patterns in a Chinese population.

## Conclusion

In summary, our findings suggest that the best clinical independent predictive factors for targeted therapy of Chinese NSCLC patients with EGFR inhibitors include an ADC histology, a nonsmoking history, and a younger age (≤65); for Chinese CRC patients, those who are female, older (>50 year old), and have ADC histology may benefit from mAb-based molecularly targeted therapies. Moreover, our designed pyrosequencing method using a novel nucleotide dispensation order is a practical and reliable method for the detection of NSCLC EGFR and CRC KRAS mutations in FFPE samples. The sensitivity, accuracy, and simplicity of the procedure are suitable for genetic testing of NSCLC and CRC patients at the clinical laboratory level.

## Additional file

Additional file 1: Table S1. Molecular epidemiological status of *EGFR* in NSCLC and *KRAS* mutation in CRC; **Table S2.** Characteristics of the human cell lines used for validation and sensitivity testing of the pyrosequencing analysis for *EGFR* and *KRAS* mutation detection; **Table S3.** Characteristics of the known mutant human NSCLC- and CRC-FFPE tissues used for validation of the designed pyrosequencing analysis; **Table S4.** Primer sequences; **Table S5.** Actual/theoretical percentages of mutant alleles at given dilutions of *EGFR* exon 19 c.2235_2249del15 (G>A) (H1650) and exon 21 c.2573T>G (H1975); **Table S6.** Actual/Theoretical Percent Mutant Allele at Given Dilutions of *KRAS* exon 2 c.35 G>T (SW480) and c.38 G>A (DLD-1); **Figure S1.** Nucleotide sequences and novel dispensation order for *EGFR* exons 18, 19, 20, and 21 mutation analysis as well as *KRAS* exon 2 mutational analysis; **Figure S2.** Analytical sensitivity for mutation detection of homozygous *KRAS* exon 2 c.35G>T on three consecutive days; **Figure S3.** Analytical sensitivity for mutation detection of heterozygous *KRAS* exon 2 c.38G>A on three consecutive days; **Figure S4.** Analytical sensitivity for in-frame deletion mutation detection of homozygous *EGFR* exon 19 c.2235_2249 del 15 (G>A) on three consecutive days; **Figure S5.** Analytical sensitivity for mutation detection of heterozygous *EGFR* exon 21 c.2573T>G on three consecutive days; **Figure S6.** Representative results of *EGFR* exons 19–21 pyrosequencing analysis in FFPE samples; **Figure S7.** Representative results of pyrosequencing *KRAS* analyses of exon 2 codons 12/13 in FFPE samples.
