# Supplementary material for: The mutation rates of EGFR in non-small cell lung cancer and KRAS in colorectal cancer of Chinese patients as detected by pyrosequencing using a novel dispensation order
Source: J Exp Clin Cancer Res. 2015 Jun 18;34(1):63. doi: 10.1186/s13046-015-0179-9 (PMC4481110; doi:10.1186/s13046-015-0179-9)
Supplement: Additional file 1: Table S1. — Molecular epidemiological status of EGFR in NSCLC and KRAS mutation in CRC; Table S2. Characteristics of the human cell lines used for validation and sensitivity testing of the pyrosequencing analysis for EGFR and KRAS mutation detection; Table S3. Characteristics of the known mutant human NSCLC- and CRC-FFPE tissues used for validation of the designed pyrosequencing analysis; Table S4. Primer sequences; Table S5. Actual/theoretical percentages of mutant alleles at given dilutions of EGFR exon 19 c.2235_2249del15 (G>A) (H1650) and exon 21 c.2573T>G (H1975); Table S6. Actual/Theoretical Percent Mutant Allele at Given Dilutions of KRAS exon 2 c.35 G>T (SW480) and c.38 G>A (DLD-1); Figure S1. Nucleotide sequences and novel dispensation order for EGFR exons 18, 19, 20, and 21 mutation analysis as well as KRAS exon 2 mutational analysis; Figure S2. Analytical sensitivity for mutation detection of homozygous KRAS exon 2 c.35G>T on three consecutive days; Figure S3. Analytical sensitivity for mutation detection of heterozygous KRAS exon 2 c.38G>A on three consecutive days; Figure S4. Analytical sensitivity for in-frame deletion mutation detection of homozygous EGFR exon 19 c.2235_2249 del 15 (G>A) on three consecutive days; Figure S5. Analytical sensitivity for mutation detection of heterozygous EGFR exon 21 c.2573T>G on three consecutive days; Figure S6. Representative results of EGFR exons 19–21 pyrosequencing analysis in FFPE samples; Figure S7. Representative results of pyrosequencing KRAS analyses of exon 2 codons 12/13 in FFPE samples. [file 13046_2015_179_MOESM1_ESM.doc]

**Supporting Information**

**
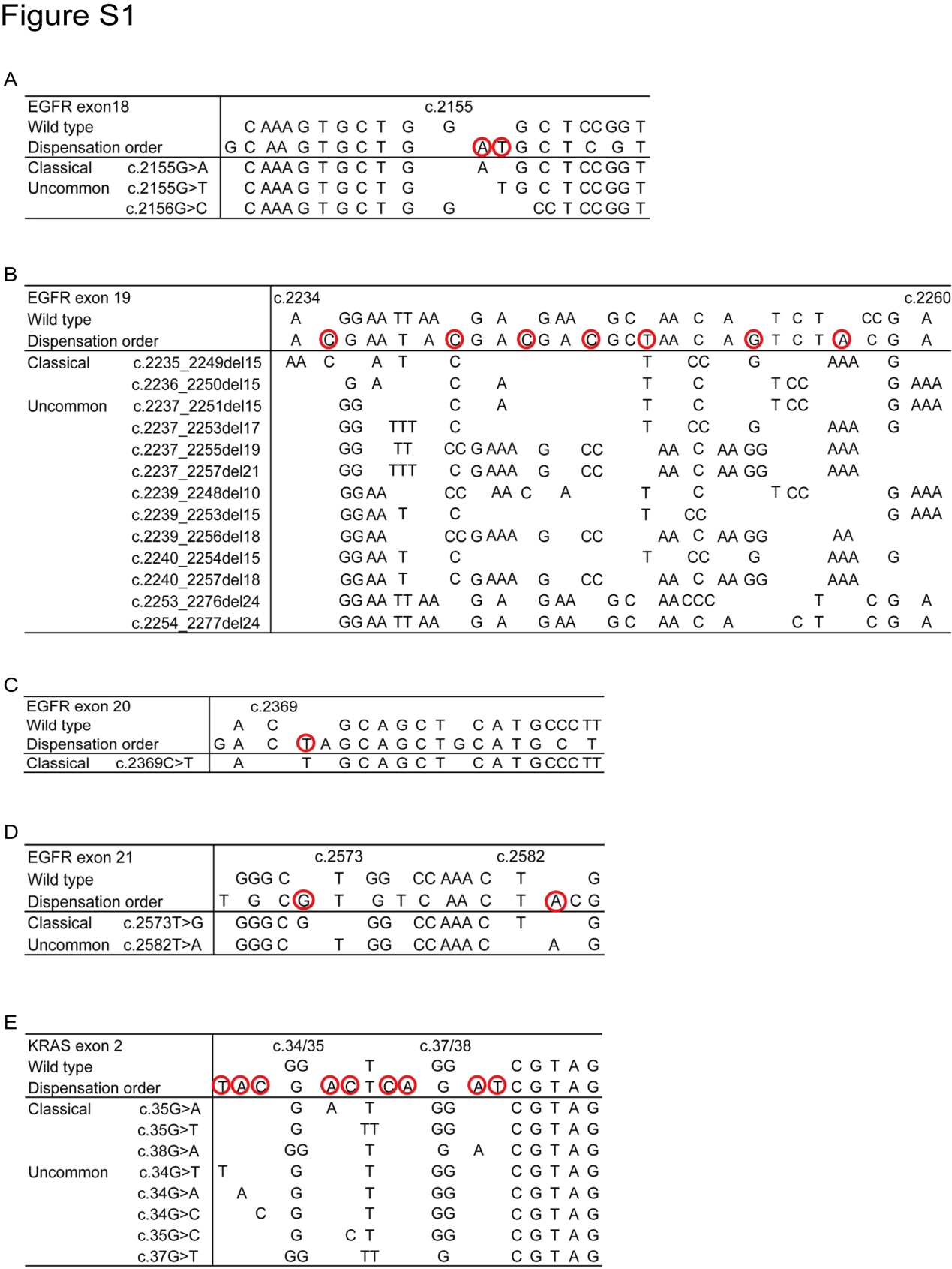
**Figure S1. Nucleotide sequences and novel dispensation order for *EGFR* exons 18 (A), 19 (B), 20 (C), and 21 (D) mutation analysis as well as *KRAS* exon 2 (E) mutational analysis. In the novel dispensation order, a number of nucleotides are present to characterize a wider group of mutated or deleted nucleotides (classical and uncommon), more than commercial kits. Circles indicate possible anomalous nucleotides identified by pyrosequencing analysis. A, adenine; G, guanine; T, thymine; C, cytosine.

**
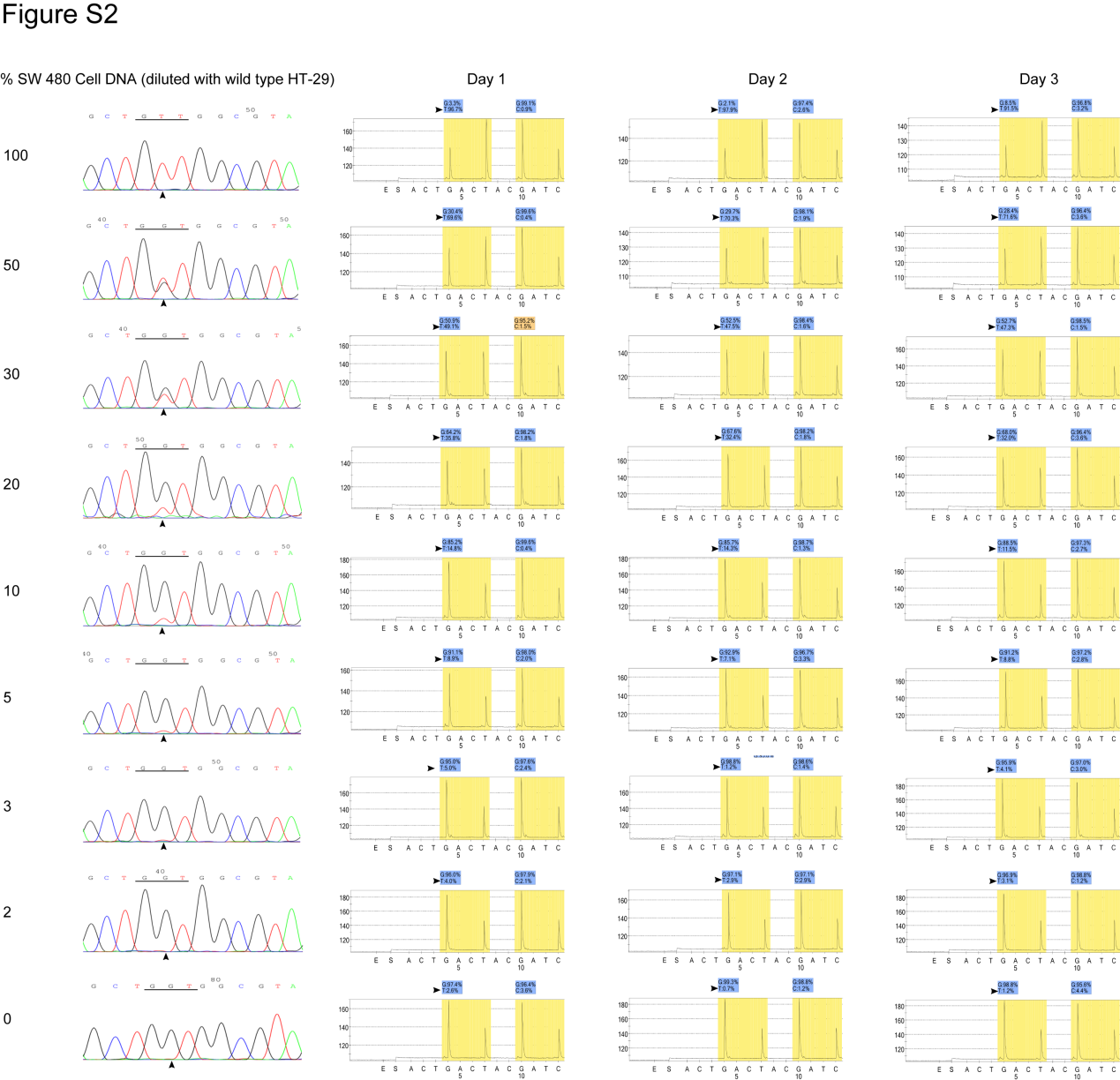
**

Figure S2. Analytical sensitivity for mutation detection of homozygous *KRAS* exon 2 c.35G>T on three consecutive days. Genomic DNA (50 ng) was used as a template. Genomic DNA harboring heterozygous or homozygous mutations was serially diluted with the corresponding wild-type DNA. The proportions of mutant DNA were adjusted to 100%, 50%, 30%, 20%, 10%, 5%, 3%, 2%, and 0% (mutant type:wild type). Pyrograms generated from the dilution series for three separate experiments (day 1, day 2, and day 3) are shown with the corresponding dideoxy sequence tracings for day 1 data. The percentage of tumor DNA corresponding to each data set is indicated at the left. Arrows indicate the mutant allele. Percentages indicate the proportion of DNA from a mutant tumor relative to DNA from a wild-type tumor.


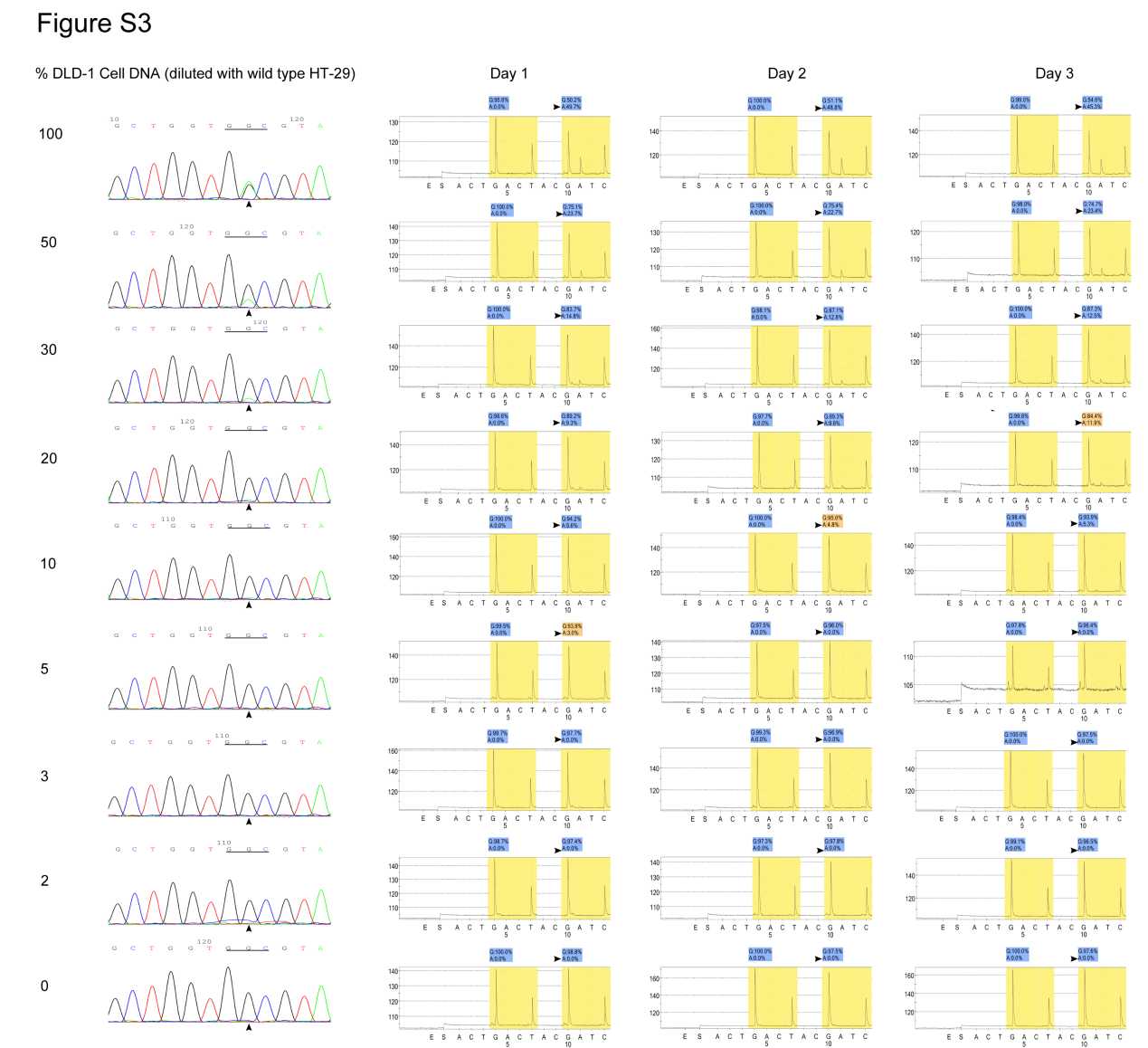


Figure S3. Analytical sensitivity for mutation detection of heterozygous *KRAS* exon 2 c.38G>A on three consecutive days. Arrows indicate the mutant allele.


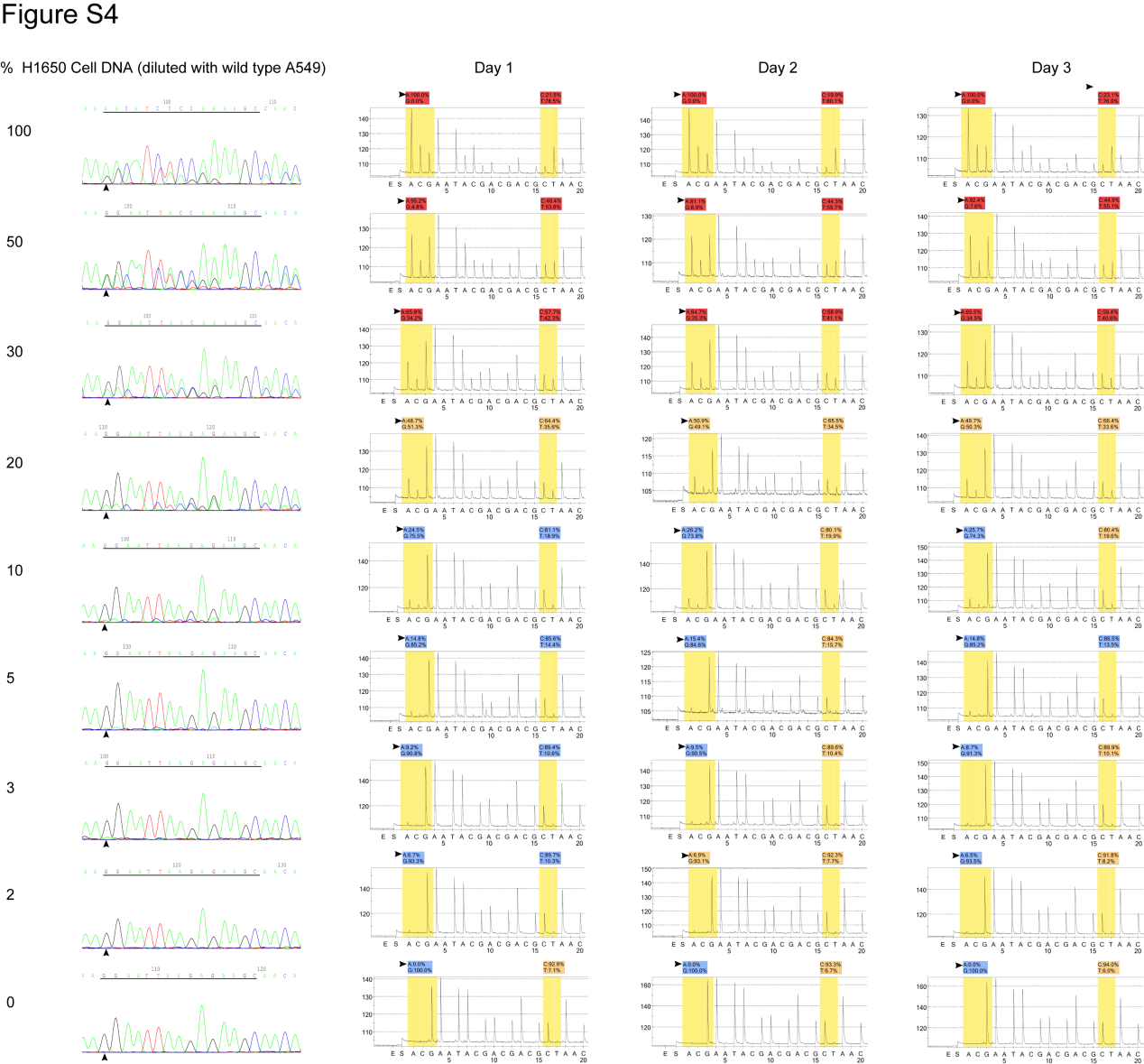


Figure S4. Analytical sensitivity for in-frame deletion mutation detection of homozygous *EGFR* exon 19 c.2235_2249 del 15 (G>A) on three consecutive days. Arrows indicate the mutant allele.


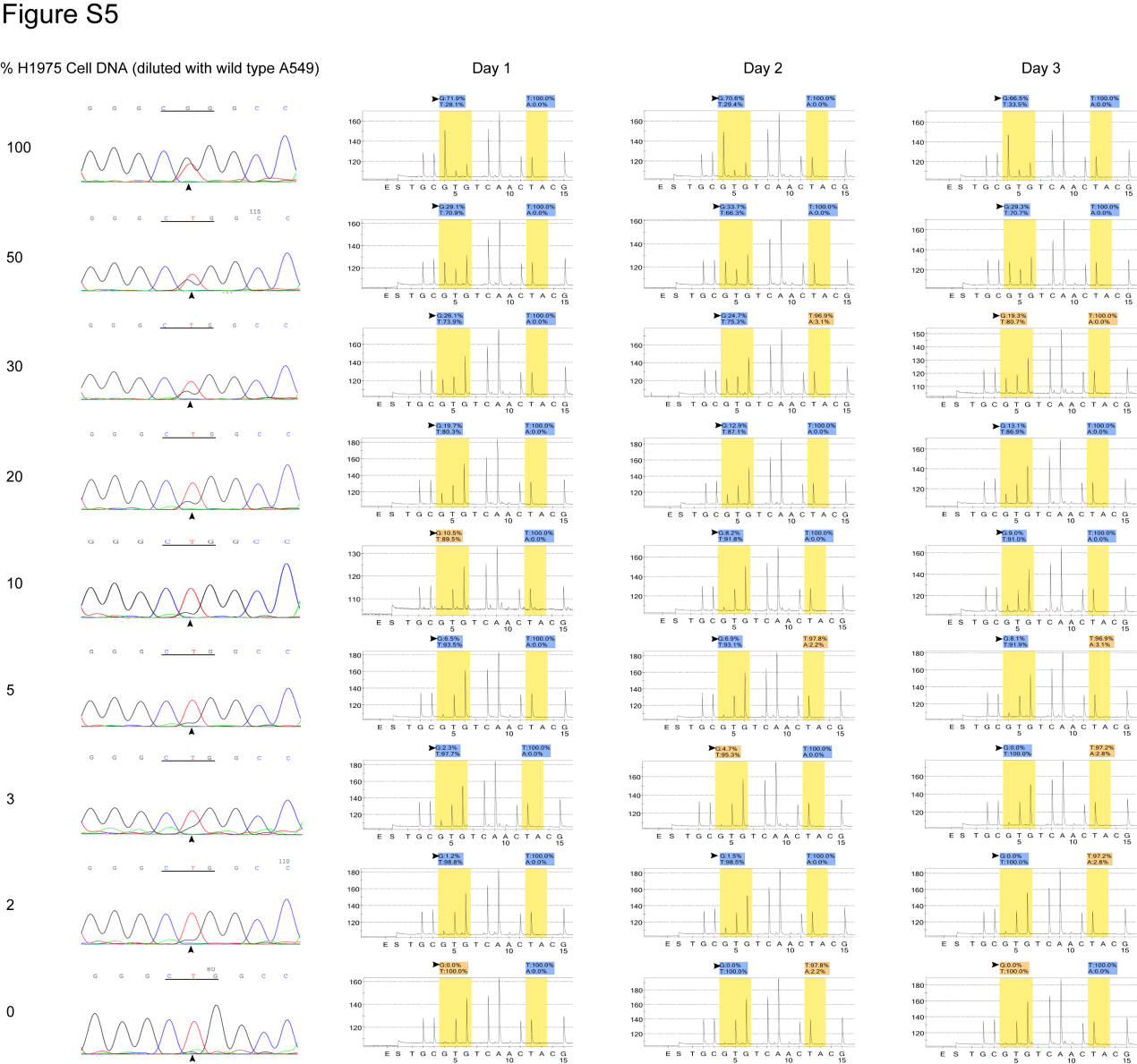


Figure S5. Analytical sensitivity for mutation detection of heterozygous *EGFR* exon 21 c.2573T>G on three consecutive days. Arrows indicate the mutant allele.


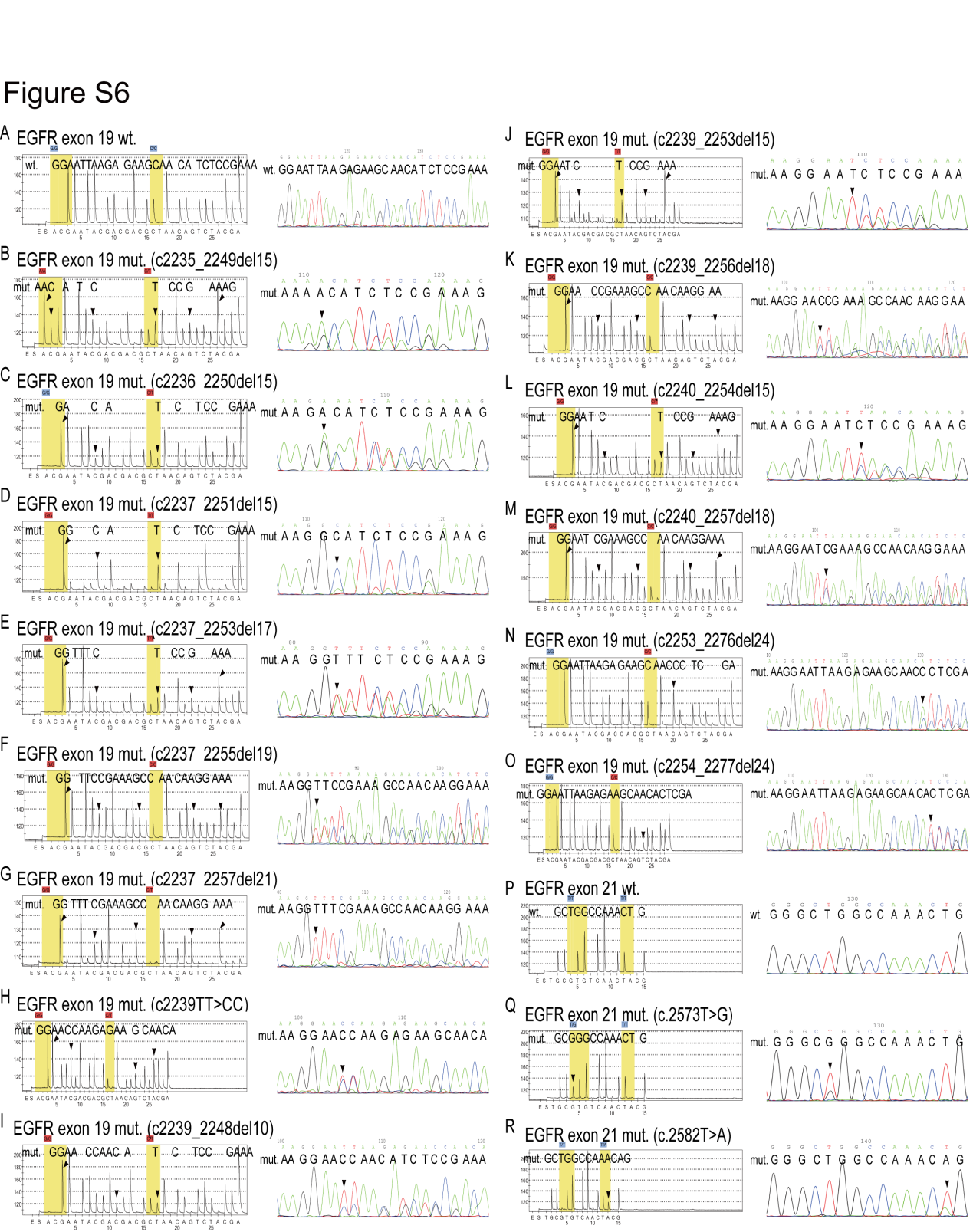


Figure S6. Representative results of *EGFR* exons 19–21 pyrosequencing analysis in FFPE samples (A–R). *EGFR* exon 19 wild-type (A). *EGFR* exon 21 wild-type (P). Dideoxy sequencing was used to confirm the pyrosequencing results. The detected nucleotides are labeled in each pyrogram and the corresponding Sanger chromatograms. Arrows indicate the mutant allele.


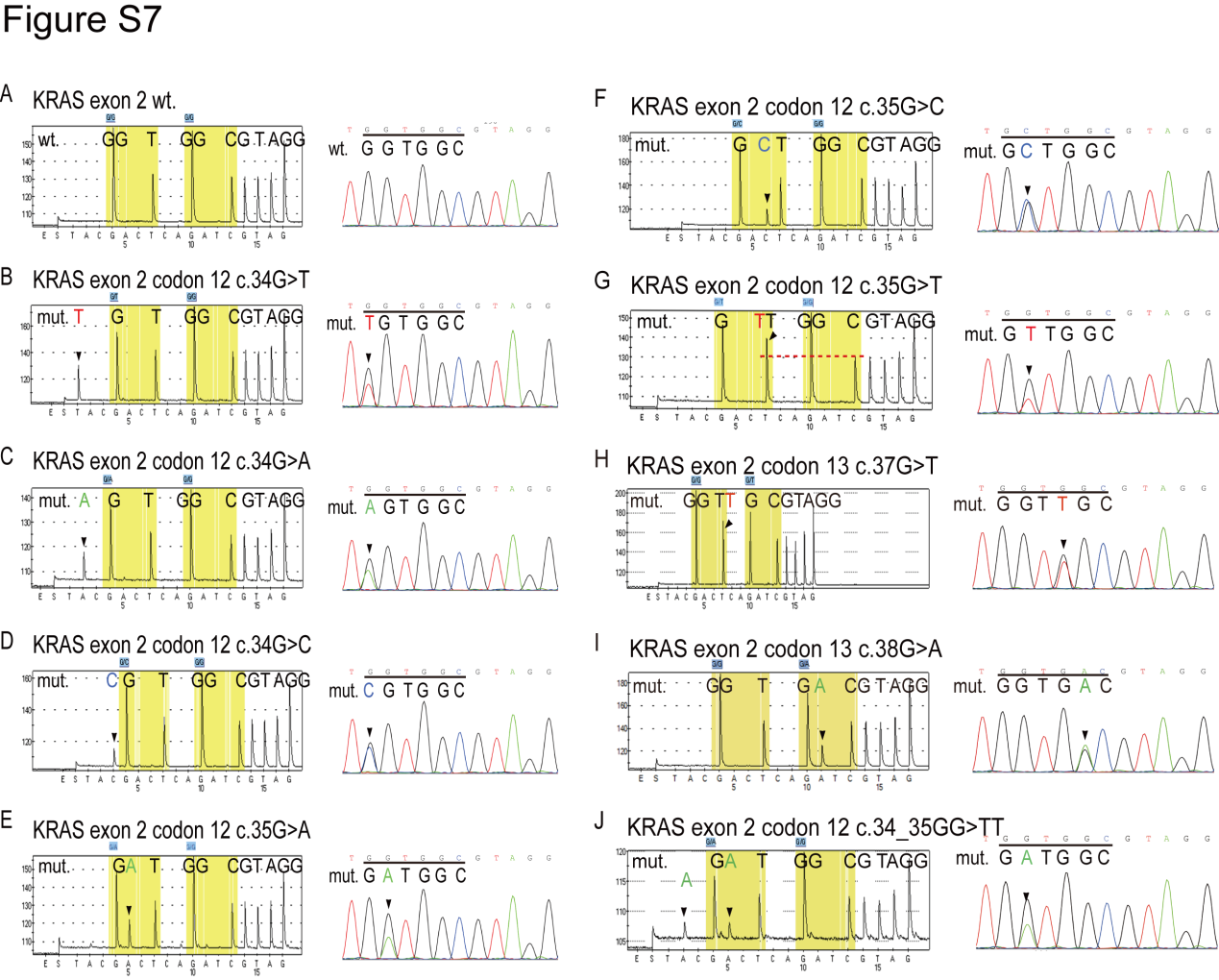


Figure S7. Representative results of pyrosequencing *KRAS* analyses of exon 2 codons 12/13 in FFPE samples (A–J). *KRAS* codon 12/13 wt (A). Dideoxy sequencing was used to confirm the pyrosequencing results. The mutation sites assayed are underlined. The detected nucleotides are labeled in each pyrogram and the corresponding Sanger chromatograms. Arrows indicate the mutant allele.

| **Table S1.** Molecular epidemiological status of *EGFR* in NSCLC and *KRAS* mutation in CRC (adapted from [9, 12]) | | | |
| --- | --- | --- | --- |
|
| Region | *EGFR* mutation rate | Region | *KRAS* mutation rate |
| Vietnam | 64.2% | Taiwan | 27% |
| Taiwan | 62.1% | Thailand | 23% |
| Thailand | 53.8% | India | 24% |
| Philippines | 52.3% | South Korea | 27% |
| Hong Kong | 47.2% | Japan | 38% |
| India | 22.2% | Oman | 31% |
| South Korea | 29% | Netherland | 37% |
| Japan | 27% | Germany | 39% |
| Australia | 7% | Australia | 28% |
| USA | 14% | USA | 43% |
| China (Guangzhou) | 19% | China (Guangzhou) | 19.7% |
| China (Beijing) | 55.8% | China (Hong Kong) | 62% |
| *EGFR*, epidermal growth factor receptor; *KRAS*, v-Ki-ras2 Kirsten rat sarcoma viral oncogene homolog; NSCLC, non-small cell lung cancers; CRC, colorectal cancer. | | | |

| **Table S2.** Characteristics of the human cell lines used for validation and sensitivity testing of the pyrosequencing analysis for *EGFR* and *KRAS* mutation detection [1,2] | | | | | |
| --- | --- | --- | --- | --- | --- |
|
| Cell line | Tissue | Gene/Exon | Mutation (nt) | Zygosity | Mutation (aa) |
| NCI-H1650 | NSCLC | *EGFR*/19 | c.2235-2249del15 | Heterozygous | p.E746_A750del5 |
| NCI-H1975 | NSCLC | *EGFR*/20 | c.2369C>T | Heterozygous | p.T790M |
|  |  | *EGFR*/21 | c.2573T>G | Heterozygous | p.L858R |
| A549 | NSCLC | *EGFR*/18-21 | wild-type |  | none |
|  |  | *KRAS*/2 | c.34G>A | Homozygous | p.G12S |
| SW480 | CRC | *KRAS*/2 | c.35G>T | Homozygous | p.G12V |
| DLD-1 | CRC | *KRAS*/2 | c.38G>A | Heterozygous | p.G13D |
| HT-29 | CRC | *KRAS*/2 | wild-type |  | none |
| *EGFR*, epidermal growth factor receptor; *KRAS*, V-Ki-ras2 Kirsten rat sarcoma viral oncogene homolog; NSCLC, non-small cell lung cancer; CRC, colorectal cancer. | | | | | |

References

1 Simonetti S, Molina MA, Queralt C, et al. Detection of EGFR mutations with mutation-specific antibodies in stage IV non-small-cell lung cancer. J Transl Med 2010, 8:135.

2 Lee J, Lee I, Han B, et al. Effect of simvastatin on cetuximab resistance in human colorectal cancer with KRAS mutations. J Natl Cancer Inst 2011,103:674-88.

| **Table S3**. Characteristics of the known mutant human NSCLC- and CRC-FFPE tissues used for validation of the designed pyrosequencing analysis | | | | | | |
| --- | --- | --- | --- | --- | --- | --- |
|
| Case | Gender | Age | Pathology | Differentiation | LN metastasis | Mutation status |
| NSCLC M1 | Female | 73 | ADC | Moderate | Yes | c. 2235_2249 del15 |
| NSCLC M2 | Male | 53 | ADC | Moderate | Yes | c. 2573 T>G |
| CRC M1 | Female | 57 | ADC | Moderate | No | c. 35 G>A |
| CRC M2 | Male | 66 | ADC | Moderate | Yes | c. 38 G>A |
| NSCLC, non-small cell lung cancers; CRC, colorectal cancer; FFPE, formalin-fixed paraffin-embedded; ADC, adenocarcinoma; LN, lymph node. | | | | | | |

| **Table S4. Primer sequences** | | | | | |
| --- | --- | --- | --- | --- | --- |
|  | | | | | |
| **Gene** | **Amplicons** | **Primer sequences** **(5ʹ to 3ʹ)** | **Ta (°C )** | **Amplicon size (bp)** |  |
|  |  | Pyrosequencing |  |  |  |
| EGFR | Exon 18 | TTGTCCCCCCCAGCTTGT (*F*) | 56 | 154 |  |
|  |  | Biotin-CTGTGCCAGGGACCTTACCTTAT (*R*) |  |
|  |  | GAAACTGAATTCAAAAAGAT (*PS*) |  |  |  |
|  | Exon 19 | GCCAGTTAACGTCTTCCTTCTCTC (*F*) | 56 | 157 |  |
|  |  | Biotin-CCACACAGCAAAGCAGAAACTC (*R*) |  |
|  |  | TCCCGTCGCTATCAA (*PS*) |  |  |  |
|  | Exon 20 | CTGGGCATCTGCCTCACCT (*F*) | 56 | 86 |  |
|  |  | Biotin-TTGTGTTCCCGGACATAGTCCA (*R*) |  |
|  |  | ACCGTGCARCTCATC (*PS*) |  |  |  |
|  | Exon 21 | GGTGAAAACACCGCAGCATGT (*F*) | 56 | 91 |  |
|  |  | Biotin-GCCTCCTTCTGCATGGTATTCT (*R*) |  |
|  |  | AAGATCACAGATTTTGG (*PS*) |  |  |  |
| KRAS | Exon 2 | GGCCTGCTGAAAATGACTGA (*F*) | 56 | 124 |  |
|  |  | Biotin-CCTCTATTGTTGGATCATATTCGTC (*R*) |  |
|  |  | TTGTGGTAGTTGGAGCT (*PS*) |  |  |  |
|  |  | Dideoxy sequencing |  |  |  |
| EGFR | Exon 18 | GCTGAGGTGACCCTTGTCTC (*F*) | 60 | 246 |  |
|  |  | ACAGCTTGCAAGGACTCTGG (*R*) |  |
|  | Exon 19 | GCTGGTAACATCCACCCAGA (*F*) | 60 | 247 |  |
|  |  | GAGAAAAGGTGGGCCTGAG (*R*) |  |
|  | Exon 20 | CCTCCTTCTGGCCACCATGCG (*F*) | 60 | 296 |  |
|  |  | CATGTGAGGATCCTGGCTCC (*R*) |  |
|  | Exon 21 | CGGATGCAGAGCTTCTTCCC (*F*) | 60 | 275 |  |
|  |  | AGGCAGCCTGGTCCCTGGTG (*R*) |  |
| KRAS | Exon 2 | GTTTGTATTAAAAGGTACTGGTG (*F*) | 58 | 286 |  |
|  |  | ATCTGTATCAAAGAATGGTCCT (*R*) |  |
| *F*, PCR forward primers; *R*, PCR reverse primers; *PS*, pyrosequencing primers; EGFR, epidermal growth factor receptor; KRAS, V-Ki-ras2 Kirsten rat sarcoma viral oncogene homolog; Ta, annealing temperature. | | | | |  |

| **Table S5.** Actual/theoretical percentages of mutant alleles at given dilutions of *EGFR* exon 19 c.2235_2249del15 (G>A) (H1650) and exon 21 c.2573T>G (H1975) | | | | | | | | | | | | | | | | | |
| --- | --- | --- | --- | --- | --- | --- | --- | --- | --- | --- | --- | --- | --- | --- | --- | --- | --- |
|
|  | ***EGFR* exon 19 c.2235_2249del15 (G>A)** | | | | | | | |  | ***EGFR* exon 21 c.2573T>G** | | | | | | | |
|  | % Mutant allele  (day 1) | |  | % Mutant allele  (day 2) | |  | % Mutant allele  (day 3) | |  | % Mutant allele  (day 1) | |  | % Mutant allele  (day 2) | |  | % Mutant allele  (day 3) | |
| % Cell line | Actual | Theoretical |  | Actual | Theoretical |  | Actual | Theoretical |  | Actual | Theoretical |  | Actual | Theoretical |  | Actual | Theoretical |
| 100 | 100 |  |  | 100 |  |  | 100 |  |  | 67 |  |  | 71 |  |  | 72 |  |
| 50 | 95 | 50 |  | 91 | 50 |  | 92 | 50 |  | 29 | 34 |  | 34 | 36 |  | 29 | 36 |
| 30 | 66 | 30 |  | 65 | 30 |  | 66 | 30 |  | 26 | 20 |  | 25 | 21 |  | 19 | 22 |
| 20 | 49 | 20 |  | 51 | 20 |  | 50 | 20 |  | 19 | 13 |  | 13 | 14 |  | 13 | 14 |
| 10 | 25 | 10 |  | 26 | 10 |  | 26 | 10 |  | 11 | 7 |  | 8 | 7 |  | 9 | 7 |
| 5 | 15 | 5 |  | 15 | 5 |  | 15 | 5 |  | 7 | 3 |  | 7 | 4 |  | 8 | 4 |
| 3 | 9 | 3 |  | 10 | 3 |  | 9 | 3 |  | 2 | 2 |  | 5 | 2 |  | 0 | 2 |
| 2 | 7 | 2 |  | 7 | 2 |  | 7 | 2 |  | 1 | 1 |  | 2 | 1 |  | 0 | 1 |
| 0 | 0 | 0 |  | 0 | 0 |  | 0 | 0 |  | 0 | 0 |  | 0 | 0 |  | 0 | 0 |
| *EGFR*, epidermal growth factor receptor | | | | | | | | | | | | | | | | | |

| **Table S6.** Actual/Theoretical Percent Mutant Allele at Given Dilutions of *KRAS* exon 2 c.35 G>T (SW480) and c.38 G>A (DLD-1) | | | | | | | | | | | | | | | | | |
| --- | --- | --- | --- | --- | --- | --- | --- | --- | --- | --- | --- | --- | --- | --- | --- | --- | --- |
|
|  | ***KRAS* exon 2 c.35 G>T** | | | | | | | |  | ***KRAS* exon 2 c.38 G>A** | | | | | | | |
|  | % Mutant allele  (day 1) | |  | % Mutant allele  (day 2) | |  | % Mutant allele  (day 3) | |  | % Mutant allele  (day 1) | |  | % Mutant allele  (day 2) | |  | % Mutant allele  (day 3) | |
| % Cell line | Actual | Theoretical |  | Actual | Theoretical |  | Actual | Theoretical |  | Actual | Theoretical |  | Actual | Theoretical |  | Actual | Theoretical |
| 100 | 97 |  |  | 98 |  |  | 92 |  |  | 50 |  |  | 49 |  |  | 45 |  |
| 50 | 70 | 49 |  | 70 | 49 |  | 72 | 46 |  | 24 | 25 |  | 23 | 25 |  | 23 | 23 |
| 30 | 49 | 29 |  | 48 | 29 |  | 47 | 28 |  | 15 | 15 |  | 13 | 15 |  | 13 | 14 |
| 20 | 36 | 19 |  | 32 | 20 |  | 32 | 18 |  | 9 | 10 |  | 10 | 10 |  | 12 | 9 |
| 10 | 15 | 10 |  | 14 | 10 |  | 12 | 9 |  | 6 | 5 |  | 5 | 5 |  | 5 | 5 |
| 5 | 9 | 5 |  | 7 | 5 |  | 9 | 5 |  | 3 | 3 |  | 0 | 2 |  | 0 | 2 |
| 3 | 5 | 3 |  | 1 | 3 |  | 4 | 3 |  | 0 | 2 |  | 0 | 1 |  | 0 | 1 |
| 2 | 4 | 2 |  | 3 | 2 |  | 3 | 2 |  | 0 | 1 |  | 0 | 1 |  | 0 | 1 |
| 0 | 3 | 0 |  | 1 | 0 |  | 1 | 0 |  | 0 | 0 |  | 0 | 0 |  | 0 | 0 |
| *KRAS*, v-Ki-ras2 Kirsten rat sarcoma viral oncogene homolog | | | | | | | | | | | | | | | | | |
